# Trehalose: is it a potential inhibitor of antithrombin polymerization?

**DOI:** 10.1042/BSR20190567

**Published:** 2019-06-18

**Authors:** Irene Martínez-Martínez

**Affiliations:** 1 Servicio de Hematología y Oncología Médica, Hospital Universitario Morales Meseguer, Centro Regional de Hemodonación, Universidad de Murcia, IMIB-Arrixaca, Murcia, Spain; 2 Grupo de Investigación CB15/00055, Centro de Investigación Biomédica en Red de Enfermedades Raras (CIBERER), Instituto de Salud Carlos III (ISCIII), Madrid, Spain

**Keywords:** Antithrombin, Polymerization, serpin, Trehalose

## Abstract

SERine Protease INhibitorS (Serpins) are a superfamily of proteins that are characterized by having a similar three-dimensional structure. The native conformation is not most thermodynamically stable, so polymerization is the main consequence when its stability is altered as a result of certain mutations. The polymerization of serpins has been a research topic for many years. Different mechanisms have been proposed and in the same way different compounds or strategies have been studied to prevent polymerization. A recent paper published in Bioscience Reports by Naseem et al. [*Biosci. Rep*. (2019) **5**, 39] studies the role of trehalose in the prevention of the polymerization of antithrombin, which belongs to the serpin superfamily. The main consequence of the antithrombin polymerization is the increased thrombotic risk, since antithrombin is the main inhibitor of the coagulation cascade. The authors demonstrate that trehalose is able to prevent the *in vitro* polymerization of antithrombin, under conditions in which it usually tends to polymerize, and demonstrate it by using different techniques. However, the binding site of trehalose in antithrombin should be defined by site-directed mutagenesis. On the other hand, it is not clear if all serpins polymerize *in vivo* through the same mechanism and it is also not clear if the same serpin can even polymerize through different mechanisms. Therefore, there are still doubts about the potential of trehalose or its derivatives to prevent *in vivo* antithrombin polymerization and, therefore, reduce thrombotic risk, as well as whether trehalose would be able to reduce polymerization in other serpins.

SERine Protease INhibitorS (Serpins) are a superfamily of structurally similar proteins but with diverse functions. Most serpins have the ability to inhibit serine proteases, although some inhibit caspases and cysteine proteases [1]. There are other serpins that do not exert any inhibitory function but act as molecular chaperones, in the transport of hormones or in the suppression of tumors [1–3]. Those serpins that have an inhibitory function perform a suicide inhibitory mechanism [4]. This mechanism is possible thanks to the fact that they present a great structural flexibility and that the native conformation is not the most thermodynamically stable [5]. But this structural flexibility has disadvantages, since certain mutations can alter the structural stability of the serpins causing the formation of inactive polymers [6]. In some serpins, polymerization not only reduces the amount of functional protein, but the intracellular accumulation of these polymers can result in cell death and organ failure [7]. The polymerization of serpins has been studied for many years but there is still no consensus on the polymerization mechanism [8–10]. However, defining the polymerization mechanism would help to rationally design molecules that are capable of reducing polymerization.

Antithrombin belongs to the serpin superfamily. It is the main inhibitor of the coagulation cascade and its main targets are FXa and thrombin. Therefore, antithrombin deficiency increases the risk of thrombosis significantly [11]. The congenital deficiency of antithrombin can be classified as type I or type II [12]. The intracellular polymerization of antithrombin results in type I deficiency increasing the thrombotic risk, athought in some cases it is possible to detect disulfide-linked dimers in the plasma [13]. Unlike the mutations that cause polymerization of α1-antitrypsin and that result in liver cirrhosis [14,15], the polymerization of antithrombin only results in increased risk of thrombosis. This is believed to be because antithrombin is synthesized at a concentration ten-times lower than α1-antitrypsin. Antithrombin has been used as a model for the study of the mechanism of serpin polymerization [8]. Antithrombin is a serpin with certain characteristics that differentiate it from the rest, such as the presence of three disulfide bridges, a structural domain that binds heparin, and three or four N-glycans, which are essential for its correct folding and functionality [16]. Curiously, although most of gross deletions or insertions result in type I deficiency, our group described a case in which the insertion of eight amino acids in the helix F of antithrombin resulted in the secretion of a non-functional protein but was not able to polymerize [17].

The study of molecules capable of reducing or preventing the polymerization of serpins has been carried out with different serpins [18–24], in order to then extrapolate the results to other serpins. In the study by Naseem et al. [25], the effect of trehalose on the polymerization of serpins has been carried out with antithrombin. Although the authors present an *in-silico* study on the potential interaction of trehalose with other serpins and reference their effect in the prevention of polymerization with neuroserpin [22], the effect on the polymerization of other serpins should be explored. The compounds investigated by the authors are generally well-known osmolytes, which operate by altering the structure of water around proteins. This fact combined with the very (∼1 M) high concentrations strongly support a non-specific effect for their action. This is acknowledged in the final paragraph of the Naseem et al. discussion [25], but they also propose that the interaction must occur in a hydrophobic area of the antithrombin. This must be demonstrated through site-directed mutagenesis, and thus define the residues involved and the type of interaction. Naseem et al.’s [25] paper showed using intrinsic fluorescence that trehalose stabilizes against the formation of the intermediate in the presence of GdnHCl. But they then choose a concentration of GdnHCl where they have shown that the intermediate is not populated (2 M) in an experiment using BisANS and infer that the low BisANS signal is due to shielding of a hydrophobic patch (rather than simply not being populated). They should have in fact chosen two GdnHCl concentrations at which the intermediate was equivalently populated in both the presence and absence of trehalose. Similarly, the possibility of spectroscopic effects on bisANS fluorescence (such as quenching) in the presence of high concentrations of sugars should be considered. Antithrombin has a dynamically regulated reactive center loop, whose conformation can affect kinetics of interaction/inhibition with different proteases. Therefore the changes induced by a heavily modified solvent could affect reactive center loop conformation rather than the process of RCL insertion as contended by Naseem et al. [25]. But the most important thing is to define the effect of trehalose or its derivatives *in vivo*. The authors demonstrate that trehalose reduces the polymerization of antithrombin in those conditions in which it usually polymerizes *in vitro*, such as at temperatures above 60°C, and they monitor this effect because antithrombin maintains its inhibitory function. *In vivo*, it would be necessary to establish a murine model of antithrombin deficiency caused by a mutation that induces polymerization, such as P80S [13], and see the effect of the administration of trehalose on the plasma activity of FXa and thrombin and on partial thromboplastin time. Although the authors state that the lowest concentration of trehalose that exerts an effect in the prevention of polymerization is 1 M, and higher concentrations have been used for the treatment of the oculopharyngeal muscular dystrophy therapy [26], it would be necessary to evaluate the absence of side effects at the concentrations required to observe the increase in inhibitory activity of antithrombin *in vivo*.

In summary, the paper of Naseem et al. [25] describes the potential of trehalose or molecules based on this scaffold to prevent the polymerization of antithrombin. Although more efforts should be made to obtain an efficient drug for use in patients, this is a step forward toward the ultimate goal.

## Abbreviations

BisANS: 4,4′-Dianilino-1,1′-Binapthyl-5,5′-Disulfonic acid, dipotassium salt
GdnHCl: Guanidinium chloride
RCL: reactive centre loop
Serpins: SERine Protease INhibitorS
